# A Proton Magnetic Resonance Spectroscopy Study of the Chronic Lead Effect on the Basal Ganglion and Frontal and Occipital Lobes in Middle-Age Adults

**DOI:** 10.1289/ehp.0800187

**Published:** 2009-02-09

**Authors:** Tsyh-Jyi Hsieh, Yi-Chun Chen, Chun-Wei Li, Gin-Chang Liu, Yu-Wen Chiu, Hung-Yi Chuang

**Affiliations:** 1 Department of Medical Imaging and; 2 Department of Community Medicine, Kaohsiung Medical University Hospital, Kaohsiung City, Taiwan; 3 Department of Medical Imaging and Radiation Technology, College of Health Sciences and; 4 Department of Radiology, College of Medicine, Kaohsiung Medical University, Kaohsiung City, Taiwan; 5 Department of Public Health, College of Health Sciences, and Center of Excellence for Environmental Medicine, Kaohsiung Medical University, Kaohsiung City, Taiwan

**Keywords:** bone lead KXRF, brain metabolism, lead, proton magnetic resonance spectroscopy

## Abstract

**Background:**

Lead is known to be a health hazard to the human brain and nervous system based on data from epidemiologic studies. However, few studies have examined the mechanism or biochemical changes caused by lead in the human brain, although recently some have used magnetic resonance spectroscopy (MRS) to test brain metabolism *in vivo*.

**Objectives:**

In this study, we used 3-T MRS to investigate brain metabolism in workers chronically exposed to lead and matched nonexposed controls.

**Materials:**

Methods: Twenty-two workers at a lead paint factory served as chronically exposed subjects of this study. These workers did not have any clinical syndromes. Eighteen age- and sex-matched nonexposed healthy volunteers served as controls. We measured blood and bone lead and used a 3-T MRS to measure their levels of brain *N*-acetyl aspartate (NAA), choline (Cho), and total creatine (tCr). A structural questionnaire was used to collect demographic, work, and health histories and information about their life habits.

**Results:**

All the MRS measures were lower in the lead-exposed group. Increased blood and bone lead levels correlated with declines in Cho:tCr ratios, especially in the occipital lobe, where changes in all gray, subcortical, and white matter were significant. Increases in blood and patella lead in every layer of the frontal lobe correlated with significant decreases in NAA:tCr ratios. One of the strongest regression coefficients was −0.023 (SE = 0.005, *p* < 0.001), which was found in the NAA:tCr ratio of frontal gray matter.

**Discussion:**

We conclude that chronic exposure to lead might upset brain metabolism, especially NAA:tCr and Cho:tCr ratios. Brain NAA and Cho are negatively correlated to blood and bone lead levels, suggesting that lead induces neuronal and axonal damage or loss. The most significant changes occurred in frontal and occipital lobes, areas in which previous neurobehavioral studies have shown memory and visual performance to be adversely affected by lead toxicity.

Lead has long been known to be a hazard to the human brain and nervous system based on data from epidemiologic studies (Castellino et al. 1995). Because leaded gasoline has been phased out and environmental levels of lead have been reduced, recent investigations have focused on subclinical damage and health effects of chronic exposure that do not result in the typical symptoms and signs. Typically, blood lead levels are measured. For example, studies of workers with low to moderate blood lead levels have reported abnormal electrophysiologic parameters and neurobehavioral performance (Baker et al. 1984; Chuang et al. 2000, 2005; Jeyaratnam et al. 1985; Lucchini et al. 2000). More recently, X-ray fluorescence (XRF) is being used to non-invasively measure the accumulation of lead in bones. Often the tibia and the patella are studied (Hu et al. 1995, 1998). Lead accumulation in the tibia, a cortical bone, can serve as a biomarker of cumulative lead dose, and there lead has a residual half-life of 25–30 years. Lead accumulation in the patella, a trabecular bone, has a half-life clearance ranging from months to years and is considered to be a biomarker for both accumulation and short-term bioavailability.

Identifying such subclinical changes in workers chronically exposed to lower levels of lead is important because brain damage occurs slowly. Neurobehavioral tests, which are now the method of determining whether lead exposure has affected brain functioning (Baker et al. 1984; Chuang et al. 2005; Lucchini et al. 2000), are somewhat slow because they measure an outcome of brain damage. Few studies have examined the mechanism or biochemical changes caused by lead in the human brain, although recently some have used magnetic resonance spectroscopy (MRS) to test brain metabolism *in vivo* (Meng et al. 2005; Trope et al. 1998, 2001; Weisskopf et al. 2004, 2007a).

MRS is a noninvasive method of examining the biochemical aspects of neurologic disease *in vivo*. The development of spatially localized spectroscopic methods that sample the relative levels of metabolites from the volume of tissues defined from magnetic resonance imaging (MRI) scanning has provided the basis for integrating the biochemical information with anatomical information obtained from the MRI (Cox 1996; Ross et al. 2006). MRS has been used to assess both neuronal viability and demyelination (Burlina et al. 2000; Ross et al. 2006). Although its use for the study of neurologic diseases has grown rapidly over the past decade, its use for investigating environmental insult to human brains is quite new (Weisskopf et al. 2007a).

Because brains of children are very vulnerable to lead exposure (Castellino et al. 1995), the brain metabolites of children exposed to high levels of lead have been found by MRS studies to have abnormalities (Meng et al. 2005; Trope et al. 1998, 2001). Recently, the Veterans Normative Aging Study, a community-based cohort of elderly U.S. men, reported an association between bone lead and increased myoinositol-to-creatine ratio in 31 people with a mean age of 77 years (Weisskopf et al. 2007a). However, to our knowledge, little research has used this approach on highly exposed adults such as lead workers. Thus, in this *in vivo* study we used MRS as well as traditional measures of lead levels and a survey, to study the effects of long-term lead exposure on brain metabolism in a group of lead workers and age- and sex-matched nonexposed controls.

## Materials and Methods

### Participants

We conducted this study in a lead paint factory, where workers were required by law to receive annual health examinations. We enrolled 22 lead-exposed workers and 18 healthy volunteers. The lead workers came from a paint factory that had 30 employees, and we included all eligible lead-exposed workers, except one female who was pregnant and seven employees who worked in business and sales and were not present in the factory during the examination days. The 18 nonexposed healthy controls volunteered when we posted an open announcement in the same area but at different plants in industries that did not use lead; thus, their socioeconomic status was similar to the lead workers. We excluded all participants with a history of alcoholism, drug abuse, and major neurologic disorders, such as severe head injury, stroke, or epilepsy. The protocol for this study was approved by the Institutional Review Board of Kaohsiung Medical University, and we obtained informed consent from each participant.

### Blood and bone lead measurements

On the same day as the MRI study, we took blood samples, measured bone lead levels, and administered the questionnaires. The blood samples were obtained by venipuncture and analyzed for whole-blood lead levels using a Zeeman-effect graphite furnace atomic absorption spectrometer (5100 PC with AS 60 autosampler; Perkin-Elmer, Waltham, MA, USA) in the same laboratory. For intralaboratory quality control, standard commercial materials (Betherning Institute, Bio-Rad; Hercules, CA, USA) were used, and all coefficients of variation were < 3%.

We examined the bone lead levels at two anatomic sites, the right middle tibia and the right patella, *in vivo* using K-shell XRF (KXRF) (Canberra Industries, Inc., Meriden, CT, USA). The middle tibia and patella were representative of primarily cortical and trabecular bones, respectively. Cortical and trabecular bones have different kinetics with respect to bone lead, which probably reflects differing architectures and bone dynamics. This phenomenon has been demonstrated directly by the differences in bone uptake and release of lead over time in follow-up KXRF studies comparing the two bone types (Tsaih et al. 2001). This painless, noninvasive measurement was performed for 30 min at each skeletal location. Radiation dosimetry studies of dose to skin, bone red marrow, and the pelvic area have demonstrated that a typical ^109^Cd KXRF measurement gives extremely low effective dose values of < 1 microsievert (μSv), significantly below the proposed limit of negligibility of 10 μSv (Hu et al. 1998; Somervaille et al. 1988; Todd et al. 1992).

On the same day, the participants answered questionnaires that contained items regarding sex, age, health status, occupation, educational level, body mass index (BMI), and lifestyle habits, including smoking, drinking, and the chewing of betel nut.

### MRI and MRS

We examined all patients using a 3.0-T whole-body magnetic resonance system (General Electric Company, Milwaukee, WI, USA) with a 40 mT/m maximum gradient capability. A head coil for conventional MRI and proton MRS was used. To evaluate the anatomic structures, before they underwent proton MRS, both the patient and control groups underwent conventional magnetic resonance protocols, including fast spin-echo T2-weighted (T2W) axial images [repetition time (TR), 4,000 msec; echo time (TE), 102 msec; field of view (FOV), 240 mm × 240 mm; thickness, 5 mm], fluid-attenuated inversion recovery (FLAIR) T2W axial images (TR, 8,627 msec; TE, 157.5 msec; inversion time, 2,100 msec; FOV, 240 mm × 240 mm; thickness, 5 mm), diffusion-weighted images (TR, 8,000 msec; TE, 71.7 msec; FOV, 240 mm × 240 mm; thickness, 5 mm; b-value, 1,000) with apparent diffusion coefficient (ADC) and enhanced ADC mapping, and FLAIR T1-weighted axial images (TR, 1,800 msec; TE, 9.8 msec; inversion time, 2,100 msec; FOV, 240 mm × 240 mm; thickness, 5 mm).

**Figure 1:**
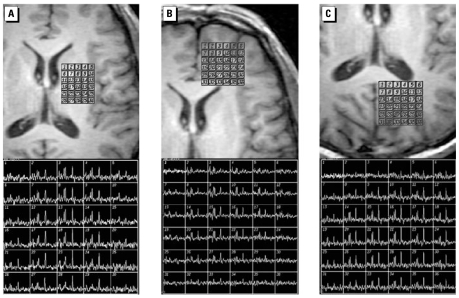
The measurement of MRS *in vivo*. (*A*) Basal ganglion. (*B*) Frontal lobe. (*C*) Occipital lobe.

We used a two-dimensional MRS imaging sequence with point-resolved spectroscopy (PRESS) volume preselection. The slice (thickness 15 mm) was transverse oriented. We examined three regions: frontal lobe, occipitoparietal lobe, and basal ganglia regions (Figure 1). Position and size of the PRESS box with outer volume suppressed were adjusted to partly exclude subcutaneous fat without excluding brain tissue. Four additional saturation bands were used to reduce string lipid signals from outside the volume of interest (VOI). We accomplished water suppression using three preceding chemical shift selective saturation (CHESS) pulses. Field homogeneity was optimized automatically over the selected VOI by observing the water signal. Spectra were acquired with TR 1,500 msec, TE 144 msec, and a matrix size of 16 × 16 over an FOV of 16 cm, resulting in a scan time of 6.4 min for each region.

We performed postprocessing of the MRS imaging data sets using the Functool software package version 2.2.49 (General Electric Company). Spectral data sets were zero filled to 1,024 points, multiplied by a 1.25-Hz Lorentzian function, and Fourier transformed in a time domain and in two spatial domains. Marquardt curve fitting was performed by using a Gaussian line shape to calculate the area under the peak for the metabolites *N*-acetyl aspartate (NAA), total creatine (tCr), and choline (Cho). We calculated the peak area ratios of Cho:tCr and NAA:tCr.

One trained rater, who was blind to the subject group status when the measurements were performed, conducted the choice of the voxels in all areas. Three voxels in each location, including gray matter, subcortical white matter, and deep white matter of the frontal lobes, the occipital lobes, and the basal ganglia, were carefully chosen. We used mean values for analysis.

### Statistical analyses

To summarize data, we used descriptive statistics to calculate the means of the continuous variables age, working duration (in years), BMI, blood lead levels, and bone lead levels. We used descriptive rates and proportions for categorical variables such as sex and uses of tobacco, alcohol, and betel nut. *t*-Tests were used to compare differences in continuous variables between the two groups, and the chi-square or Fisher exact tests were used to test differences in nominal variables between the two groups. Multiple linear regressions were used to test the association between each MRS measure and each lead parameter, with adjustment for sex, age, and smoking status. Because the units of blood and bone lead levels were different, we compared the effect using standardized regression coefficients. All statistics operations were performed with SPSS software (version 14.0.0; SPSS, Inc., Chicago, IL, USA). *p*-Values < 0.05 (two-tailed) were considered significant.

## Results

Table 1 shows all the blood and bone (tibia and patella) lead levels. The mean ± SD of blood and bone lead levels in exposed group were 16.99 ± 10.38 μg/dL, 61.55 ± 30.21 μg/g tibia bone minerals, and 66.29 ± 19.48 μg/g patella bone minerals, respectively, and in the nonexposed controls, 3.4 ± 1.11 μg/dL, 18.51 ± 22.4 μg/g tibia bone minerals, and 7.14 ± 9.81 μg/g patella bone minerals, respectively. The Pearson correlation coefficients were 0.588 (*p* < 0.001) between patella and tibia lead, 0.685 (*p* < 0.001) between patella and blood, and 0.544 (*p* < 0.001) between tibia and blood lead levels.

The exposure and nonexposure groups had similar age, BMIs, and life habits. Although the study group had a higher prevalence of smoking (45.4%), alcohol drinking (13.6%), and betel nut chewing (13.6%), the differences by chi-square tests were insignificant.

Both the lead-exposed and nonexposed participants had normal neurologic imaging findings and no clinical evidence of neural diseases. The frontal gray and white matter of the lead-exposed group had a significantly lower Cho:tCr ratio than the nonexposed group. In the occipital lobe, all Cho:tCr ratios of gray, subcortical, and white matter in the lead-exposed group were significantly lower than those of the nonexposed group. That ratio in basal ganglions was also lower in the exposed group than in those measured in the nonexposed group, although not significant. Similarly, all the frontal lobe NAA:tCr ratios in the exposed group were lower than those of the nonexposed group. In the occipital lobe, only white matter in the exposed group had significantly lower NAA:tCr ratios compared with those of nonexposed. In the basal ganglion, the NAA:tCr ratios of those exposed to lead were significantly lower than those found in the controls (Table 2).

Table 3 shows the association between human brain MRS measurements and lead biomarkers (including blood, patella, and tibia lead) with 42 multiple regressions. The dependent variables were the MRS measurements; the main predictors of interest to us were the lead biomarkers (blood, patella, and tibia lead levels), adjusting for sex, age, and smoking status (yes vs. no). Because the units of blood and bone lead levels were different, we compared effects using standardized regression coefficients in Table 3. Decreases in Cho:tCr ratios were significantly associated with increases in blood and bone lead levels, especially in the occipital lobe where all gray, subcortical, and white matter values were significant. These data suggest that Cho and Cho-containing compounds in the occipital lobe, compared with the other areas of brain, were the most sensitive to all blood and bone lead levels in these workers. However, the NAA:tCr ratios in every layer of the frontal brain were significantly inversely associated with blood and patella lead levels, suggesting that NAA in the frontal lobe might be the most sensitive to recent and subcumulative lead exposure. One of the strongest regression coefficients was −0.023 (SE = 0.005, *p* < 0.001, standardized coefficient = −0.764) for the NAA:tCr ratio in frontal gray matter and blood lead.

## Discussion

This MRS study comparing the brain metabolism of middle-age workers chronically exposed to lead with matched nonexposed controls revealed a negative correlation between blood and bone lead and brain NAA and Cho levels, especially in the frontal and occipital lobes.

The effects of lead toxicity have been examined in the context of neurobehavioral evaluations (Balbus-Kornfeld et al. 1995; Chuang et al. 2005; Shih et al. 2007). Although the adverse effect of lead exposure on neurobehavioral functioning is one of the most consistently reported impairments associated with lead exposure, little is known about the effects of lead on brain metabolism *in vivo* or about the structural and functional correlates of lead-related brain dysfunction (Seeber et al. 2002). Thus, there has been a growing interest in the mechanisms through which lead affects brain function.

MRS is used widely to assess both neuronal viability and demyelination and can detect both NAA and creatine in discrete tissue volumes. A decrease in NAA, which is located in neuronal cell bodies, has been proposed to indicate possible neuronal and axonal damage or loss, and, practically, the decrease in NAA is measured relative to the level of creatine, a stable metabolite whose level is constant after neuronal loss (van der Knaap et al. 1992). In the present study, we found a significant decline in NAA:tCr ratio in the presence of increased blood and patella lead levels in both the gray and white matter in the frontal lobe, an area of the brain that plays a critical role in the retrieval of source information. The memory component in the frontal lobe is more restricted to working memory, which is a component of executive function. This finding may help explain the lower memory task in neurobehavioral performance in lead workers (Chuang et al. 2005; Schwartz et al. 2000, 2005; Seeber et al. 2002; Shih et al. 2007).

In the present study, the intensity of the Cho MRS signal was also reduced with increases in all blood and bone lead measures in the occipital lobe. The occipital lobe is the visual processing center of the mammalian brain and contains most of the anatomical region of the visual cortex. Lead exposure and exposed workers have indeed been found to have poor visuospatial/visuomotor domain processing on neurobehavioral tests (Schwartz et al. 2000, 2005; Seeber et al. 2002; Shih et al. 2007; Weisskopf et al. 2007b). A low Cho signal may indicate decreased cell membrane turnover or myelin alterations that can lead to brain shrinkage (Cox 1996; Ross et al. 2006). The findings of reduced Cho:tCr ratios in the exposure group were similar to the findings in the hepatic encephalopathy that may alter transport of Cho, decrease nutritional uptake, and impair brain metabolism (Zwingmann 2007). These previous studies suggest that lead-induced neurotoxicity might alter the neural transmitters, although underlying mechanisms are not clear. Changes of the neural transmitters may be one of the possible reasons for the reduction in Cho:tCr ratios.

In our study, reduced NAA:tCr and Cho:tCr ratios in the basal ganglia, the cortical gray matter, the subcortical white matter, and the deep white matter suggests that lead-induced encephalopathy affects the gray matter and the white matter in a similar manner and that closer attention should be paid to systemic influences. Reductions in the cortical gray matter and the subcortical white matter, both high-function areas of the brain, may explain at least in part the impaired cognitive functions previously reported in relation to lead exposure. The previous MRI studies suggest that progressive and irreversible changes to the brain architecture may be caused by lead exposure (Schwartz et al. 2000; Stewart et al. 2006). The white matter changes in our study are consistent with these MRI findings.

Our study has several limitations. Technically, two-dimensional MRS can provide data from a larger region than can single-voxel MRS. However, increased background noise made the myoinositol and the glutamate complex undetectable in our study. Recent improvements in hardware and software, which produce a better signal-to-noise ratio, may improve estimation in future two-dimensional MRS studies. The small sample size is also a limitation affecting the power of this study. Only 22 lead workers in a small factory and 18 nonexposed volunteers participated in the MRS measurements. An extended study with larger screening might provide more information about the association between lead and brain damage.

In summary, lead toxicity to the human brain is complex and not well recognized. Our study showed that brain metabolism, especially NAA:tCr and Cho:tCr ratios, might be disturbed by lead. Brain NAA and Cho were negatively correlated with blood and bone lead levels, suggesting that lead may induce neuronal and axonal damage or loss. The most significant regions were frontal and occipital lobes, areas previously associated with impaired memory and visual performance on neurobehavioral tests of people exposed to lead.

## Figures and Tables

**Table 1:** Characteristics of participants, and blood and bone lead levels.

Characteristic	Nonexposed (*n* = 18)	Exposed (*n* = 22)	*p*-Value
Age [years (mean ± SD)]	46.06 ± 10.14	45.71 ± 11.72	0.92^a^
Working duration [years (mean ± SD)]	—	12.95 ± 8.34	
BMI [kg/m^2^ (mean ± SD)]	24.48 ± 2.42	24.49 ± 3.07	0.99^a^
Lead			
Blood [μg/dL (mean ± SD)]	3.40 ± 1.11	16.99 ± 10.38	< 0.001^a^
Tibia [μg/g bone (mean ± SD)]	18.51 ± 22.40	61.55 ± 30.21	< 0.001^a^
Patella [μg/g bone (mean ± SD)]	7.14 ± 9.81	66.29 ± 19.48	< 0.001^a^
Sex [*n* (%)]			0.731^b^
Female	5 (27.8)	5 (22.7)	
Male	13 (72.2)	17 (77.3)	
Years of education [*n* (%)]			0.788^b^
≤ 9	4 (22.2)	7 (31.8)	
9–12	9 (50.0)	10 (45.5)	
> 12	5 (27.8)	5 (22.7)	
Smoking [*n* (%)]			0.09^b^
No	15 (83.3)	12 (54.5)	
Yes	3 (16.7)	10 (45.5)	
Drinking [*n* (%)]			> 0.99^b^
No	15 (83.3)	19 (86.4)	
Yes	3 (16.7)	3 (13.6)	
Chew betel nut [*n* (%)]			0.613^b^
No	17 (94.4)	19 (86.4)	
Yes	1 (5.6)	3 (13.6)	

a *t*-Test.

b Chi-square test.

**Table 2:** Comparisons of brain magnetic resonance between lead-exposed workers and the nonexposed group.

MRS measures	Nonexposed (*n* = 18) (mean ± SD)	Exposed (*n* = 22) (mean ± SD)	*p*-Value^a^
Cho:tCr ratio			
Frontal lobe gray matter	1.430 ± 0.312	1.159 ± 0.121	0.002
Subcortical white matter	1.292 ± 0.273	1.198 ± 0.232	0.247
White matter	1.467 ± 0.222	1.276 ± 0.329	0.042
Basal ganglion	1.026 ± 0.183	1.017 ± 0.207	0.889
Occipital lobe gray matter	1.137 ± 0.240	0.805 ± 0.290	< 0.001
Subcortical white matter	1.148 ± 0.239	0.821 ± 0.276	< 0.001
White matter	1.417 ± 0.320	1.092 ± 0.369	0.006
NAA:tCr ratio			
Frontal lobe gray matter	1.702 ± 0.314	1.390 ± 0.214	0.001
Subcortical white matter	1.566 ± 0.272	1.342 ± 0.185	0.004
White matter	1.684 ± 0.334	1.477 ± 0.297	0.046
Basal ganglion	1.107 ± 0.263	0.950 ± 0.199	0.038
Occipital lobe gray matter	1.543 ± 0.293	1.431 ± 0.405	0.331
Subcortical white matter	1.451 ± 0.237	1.290 ± 0.265	0.051
White matter	1.602 ± 0.306	1.162 ± 0.409	0.001

a *t*-Test.

**Table 3:** Regression coefficients of human brain Cho:tCr and NAA:tCr ratios influenced by blood and bone lead levels (independent variables) with adjustment of sex, age, and smoking status.

	Blood lead		Patella lead		Tibia lead	
Dependent variable	β (SE)	Sdβ^a^	β (SE)	Sdβ^a^	β (SE)	Sdβ^a^
Cho:tCr						
Frontal lobe						
Gray matter	−0.014 (0.005)*	−0.566	−0.004 (0.001)*	−0.549	−0.004 (0.001)*	−0.495
Subcortical white matter	−0.005 (0.005)	−0.187	−0.002 (0.001)	−0.203	−0.001 (0.001)	−0.100
White matter	−0.013 (0.005)*	−0.459	−0.003 (0.001)	−0.296	−0.002 (0.001)	−0.187
Basal ganglion	0.002 (0.004)	−0.086	0.005 (0.001)	−0.086	0.005 (0.001)	−0.084
Occipital lobe						
Gray matter	−0.016 (0.005)*	−0.526	−0.004 (0.001)*	−0.382	−0.004 (0.001)*	−0.423
Subcortical white matter	−0.013 (0.006)*	−0.448	−0.004 (0.001)*	−0.457	−0.005 (0.001)*	−0.546
White matter	−0.018 (0.007)*	−0.486	−0.006 (0.002)*	−0.499	−0.004 (0.002)*	−0.395
NAA:tCr						
Frontal lobe						
Gray matter	−0.023 (0.005)*	−0.764	−0.004 (0.001)*	−0.480	−0.003 (0.001)*	−0.392
Subcortical white matter	−0.011 (0.005)*	−0.451	−0.003 (0.001)*	−0.442	−0.002 (0.001)	−0.259
White matter	−0.015 (0.006)*	−0.465	−0.004 (0.002)*	−0.436	−0.001 (0.002)	−0.122
Basal ganglion	−0.005 (0.005)	−0.219	−0.002 (0.001)	−0.280	−0.001 (0.001)	−0.134
Occipital lobe						
Gray matter	−0.003 (0.007)	−0.087	−0.002 (0.002)	−0.233	−0.003 (0.002)	−0.243
Subcortical white matter	−0.001 (0.005)	−0.045	−0.002 (0.001)	−0.268	−0.001 (0.001)	−0.142
White matter	−0.018 (0.007)*	−0.425	−0.006 (0.002)*	−0.477	−0.005 (0.002)*	−0.369

a Standard regression coefficients.

* *p*< 0.05.

## References

[b1-ehp-117-941] Baker EL, Feldman RG, White RA, Harley JP, Niles CA, Dinse GE (1984). Occupational lead neurotoxicity: a behavioural and electrophysiological evaluation. Study design and year one results. Br J Ind Med.

[b2-ehp-117-941] Balbus-Kornfeld JM, Stewart W, Bolla KI, Schwartz BS (1995). Cumulative exposure to inorganic lead and neurobehavioural test performance in adults: an epidemiological review. Occup Environ Med.

[b3-ehp-117-941] Burlina AP, Aureli T, Bracco F, Conti F, Battistin L (2000). MR spectroscopy: a powerful tool for investigating brain function and neurological diseases. Neurochem Res.

[b4-ehp-117-941] Castellino N, Castellino P, Sannolo N (1995). Inorganic Lead Exposure: Metabolism and Intoxication.

[b5-ehp-117-941] Chuang HY, Chao KY, Tsai SY (2005). Reversible neurobehavioral performance with reductions in blood lead levels—a prospective study on lead workers. Neurotoxicol Teratol.

[b6-ehp-117-941] Chuang HY, Schwartz J, Tsai SY, Lee ML, Wang JD, Hu H (2000). Vibration perception thresholds in workers with long term exposure to lead. Occup Environ Med.

[b7-ehp-117-941] Cox IJ (1996). Development and applications of in vivo clinical magnetic resonance spectroscopy. Prog Biophys Mol Biol.

[b8-ehp-117-941] Hu H, Aro A, Rotnitzky A (1995). Bone lead measured by X-ray fluorescence: epidemiologic methods. Environ Health Perspect.

[b9-ehp-117-941] Hu H, Rabinowitz M, Smith D (1998). Bone lead as a biological marker in epidemiologic studies of chronic toxicity: conceptual paradigms. Environ Health Perspect.

[b10-ehp-117-941] Jeyaratnam J, Devathasan G, Ong CN, Phoon WO, Wong PK (1985). Neurophysiological studies on workers exposed to lead. Br J Ind Med.

[b11-ehp-117-941] Lucchini R, Albini E, Cortesi I, Placidi D, Bergamaschi E, Traversa F (2000). Assessment of neurobehavioral performance as a function of current and cumulative occupational lead exposure. Neurotoxicology.

[b12-ehp-117-941] Meng XM, Zhu DM, Ruan DY, She JQ, Luo L (2005). Effects of chronic lead exposure on 1H MRS of hippocampus and frontal lobes in children. Neurology.

[b13-ehp-117-941] Ross BD, Coletti P, Lin A, Edelman RR, Hesselink JR, Zlatkin MB, Crues JV (2006). Magnetic resonance spectroscopy of the brain: neurospectroscopy. Clinical Magnetic Resonance Imaging.

[b14-ehp-117-941] Schwartz BS, Lee BK, Bandeen-Roche K, Stewart W, Bolla K, Links J (2005). Occupational lead exposure and longitudinal decline in neurobehavioral test scores. Epidemiology.

[b15-ehp-117-941] Schwartz BS, Stewart WF, Bolla KI, Simon PD, Bandeen-Roche K, Gordon PB (2000). Past adult lead exposure is associated with longitudinal decline in cognitive function. Neurology.

[b16-ehp-117-941] Seeber A, Meyer-Baron M, Schaper M (2002). A summary of two meta-analyses on neurobehavioural effects due to occupational lead exposure. Arch Toxicol.

[b17-ehp-117-941] Shih RA, Hu H, Weisskopf MG, Schwartz BS (2007). Cumulative lead dose and cognitive function in adults: a review of studies that measured both blood lead and bone lead. Environ Health Perspect.

[b18-ehp-117-941] Somervaille LJ, Chettle DR, Scott MC, Tennant DR, McKiernan MJ, Skilbeck A (1988). In vivo tibia lead measurements as an index of cumulative exposure in occupationally exposed subjects. Br J Ind Med.

[b19-ehp-117-941] Stewart WF, Schwartz BS, Davatzikos C, Shen D, Liu D, Wu X (2006). Past adult lead exposure is linked to neuro-degeneration measured by brain MRI. Neurology.

[b20-ehp-117-941] Todd AC, McNeill FE, Palethorpe JE, Peach DE, Chettle DR, Tobin MJ (1992). In vivo X-ray fluorescence of lead in bone using K X-ray excitation with 109Cd sources: radiation dosimetry studies. Environ Res.

[b21-ehp-117-941] Trope I, Lopez-Villegas D, Cecil KM, Lenkinski RE (2001). Exposure to lead appears to selectively alter metabolism of cortical gray matter. Pediatrics.

[b22-ehp-117-941] Trope I, Lopez-Villegas D, Lenkinski RE (1998). Magnetic resonance imaging and spectroscopy of regional brain structure in a 10-year-old boy with elevated blood lead levels. Pediatrics.

[b23-ehp-117-941] Tsaih SW, Korrick S, Schwartz J, Lee ML, Amarasiriwardena C, Aro A (2001). Influence of bone resorption on the mobilization of lead from bone among middle-aged and elderly men: the Normative Aging Study. Environ Health Perspect.

[b24-ehp-117-941] van der Knaap MS, van der Grond J, Luyten PR, den Hollander JA, Nauta JJ, Valk J (1992). 1H and 31P magnetic resonance spectroscopy of the brain in degenerative cerebral disorders. Ann Neurol.

[b25-ehp-117-941] Weisskopf MG, Hu H, Mulkern RV, White R, Aro A, Oliveira S (2004). Cognitive deficits and magnetic resonance spectroscopy in adult monozygotic twins with lead poisoning. Environ Health Perspect.

[b26-ehp-117-941] Weisskopf MG, Hu H, Sparrow D, Lenkinski RE, Wright RO (2007a). Proton magnetic resonance spectroscopic evidence of glial effects of cumulative lead exposure in the adult human hippocampus. Environ Health Perspect.

[b27-ehp-117-941] Weisskopf MG, Proctor SP, Wright RO, Schwartz J, Spiro A, Sparrow D (2007b). Cumulative lead exposure and cognitive performance among elderly men. Epidemiology.

[b28-ehp-117-941] Zwingmann C (2007). Nuclear magnetic resonance studies of energy metabolism and glutamine shunt in hepatic encephalopathy and hyperammonemia. J Neurosci Res.

